# Regeneration of unconventional natural gas by methanogens co-existing with sulfate-reducing prokaryotes in deep shale wells in China

**DOI:** 10.1038/s41598-020-73010-6

**Published:** 2020-09-29

**Authors:** Yimeng Zhang, Zhisheng Yu, Yiming Zhang, Hongxun Zhang

**Affiliations:** 1 College of Resources and Environment, University of Chinese Academy of Sciences, 19 A Yuquan Road, Beijing, 100049 People’s Republic of China; 2 Institute of Oceanology, Chinese Academy of Sciences, Qingdao, 266071 People’s Republic of China; 3 Open Studio for Marine Corrosion and Protection, Pilot National Laboratory for Marine Science and Technology (Qingdao), No.1 Wenhai Road, Qingdao, 266237 People’s Republic of China; 4 Beijing Municipal Ecological Environment Bureau, Beijing, 100048 People’s Republic of China

**Keywords:** Microbiology, Environmental sciences

## Abstract

Biogenic methane in shallow shale reservoirs has been proven to contribute to economic recovery of unconventional natural gas. However, whether the microbes inhabiting the deeper shale reservoirs at an average depth of 4.1 km and even co-occurring with sulfate-reducing prokaryote (SRP) have the potential to produce biomethane is still unclear. Stable isotopic technique with culture-dependent and independent approaches were employed to investigate the microbial and functional diversity related to methanogenic pathways and explore the relationship between SRP and methanogens in the shales in the Sichuan Basin, China. Although stable isotopic ratios of the gas implied a thermogenic origin for methane, the decreased trend of stable carbon and hydrogen isotope value provided clues for increasing microbial activities along with sustained gas production in these wells. These deep shale-gas wells harbored high abundance of methanogens (17.2%) with ability of utilizing various substrates for methanogenesis, which co-existed with SRP (6.7%). All genes required for performing methylotrophic, hydrogenotrophic and acetoclastic methanogenesis were present. Methane production experiments of produced water, with and without additional available substrates for methanogens, further confirmed biomethane production via all three methanogenic pathways. Statistical analysis and incubation tests revealed the partnership between SRP and methanogens under in situ sulfate concentration (~ 9 mg/L). These results suggest that biomethane could be produced with more flexible stimulation strategies for unconventional natural gas recovery even at the higher depths and at the presence of SRP.

## Introduction

Shale gas, an alternative energy source for conventional oil and gas, has changed the world’s energy structure. Currently, many countries are increasing their commercial exploration of shale gas, including China. The shales in the Sichuan Basin, in southern China, host a large unconventional natural shale-gas deposit, with an estimated 2.9 trillion m^3^ of recoverable gas. Horizontal drilling and hydraulic fracturing technologies have been employed advantageously to extract economic volumes of shale gas trapped in low-permeability and deep shale rock. Despite the unique geological features such as the greater buried depth of exploration (on average 4162 m) and higher degree of thermal evolution (2.5–4%) in the shale^1^, China has successfully emploied such technologies in extracting commercial shale gas. However, concerns are arising about the rates of well decline, although well productivity was high initially. Well decline is attributed to decreased shale gas production with the prolonged period of gas extraction^2^. Accordingly, interest has been shown recently in proposed new techniques that could potentially regenerate secondary shale gas^3,4^.

Microbial generation of economic accumulations of methane within shales may make contribution to total shale gas production. Methanogenesis that mainly taken by methanogenic archaea could happen at two different periods. The first period is the initial stage of shale gas formation to form microbial gas resources before commercial extraction^5^. Additionally, methanogenesis possibly happens in hydraulically fractured shales to regenerate biomethane after hydraulic fracturing operations that change the geochemical characteristics downhole^6^. Methanogenic microorganisms in shale formations are likely stimulated as fracturing fluid provides the organic and inorganic chemical inputs (like glycine betaine, choline, sucrose and ethylene glycol) for their colonization and persistence^7^. Even though shales that have largely been attributed to thermogenic processes, they could contain far more microbial methane than previously believed^4^. Confirmation of the biogenic methane in shales with thermogenic origin is important because biogenic way of methane would mean faster regeneration than thermogenic processes. Generally, biomethane is formed by methanogens via methylotrophic (methyl compounds disproportionation), hydrogenotrophic (carbon dioxide reduction using hydrogen) and acetoclastic pathways (acetate fermentation). Diverse methanogens, such as hydrogenotrophic *Methanocalculus* and methylotrophic *Methanohalophilus*, have been identified in shale-gas produced water^4,8–10^. However, few studies are available for investigating biomethane formation mechanisms in so deep shale-gas wells (on average 4162 m). Likewise, for further biomethane stimulation, another question needs to be answered is that what is the relationship between sulfate-reducing prokaryotes (SRP) and methanogens under the in situ geochemical conditions in such deep wells?

In fact, a diverse array of SRP like *Thermotoga*, *Petrotoga* and *Desulfuromonas*, which anaerobically generate sulfide using sulfate as electron acceptors during their respiration process, have been found in fractured shale-gas wells^10–12^. For practical shale gas exploration, SRP have driven much attention for leading to reservoir souring and infrastructure corrosion in natural gas wells^13^. In addition, SRP could act as partner or competitor of methanogens due to their competition over common substrates such as hydrogen and acetate, and therefor influence methane production^14,15^. For instance, in complex natural environment such as sulfate-rich marine sediments, methanogenesis is generally restricted by sulfate reduction which has higher affinity for available substrates than methane production^16–18^.

This study, aims to explore biomethane formation pathways, as well as how SRP affect methanogenic activities under in situ geochemical conditions during deep shale-gas production (with an average depth of 4.1 km) through integrated analysis based on culture-dependent and independent methods. Gas samples were collected to analyse the stable isotope composition for determining whether the origin of shale gas was thermogenic or biogenic. Subsequently, we collected microbial biomass from filtered co-produced water in seven shale-gas wells. Microbial diversity related to methanogenic activities was examined by 16S rRNA gene sequencing of the concentrated biomass. Metagenomic sequencing was also performed for investigating possible methanogenic pathways. Further, incubation experiments lasting 110 days were conducted to confirm the methane production potential and the major methanogenic pathways as well as to explore the relationships between SRP and methanogens.

## Results

### The origin of shale gas based on stable isotopic ratios

**Figure 1:**
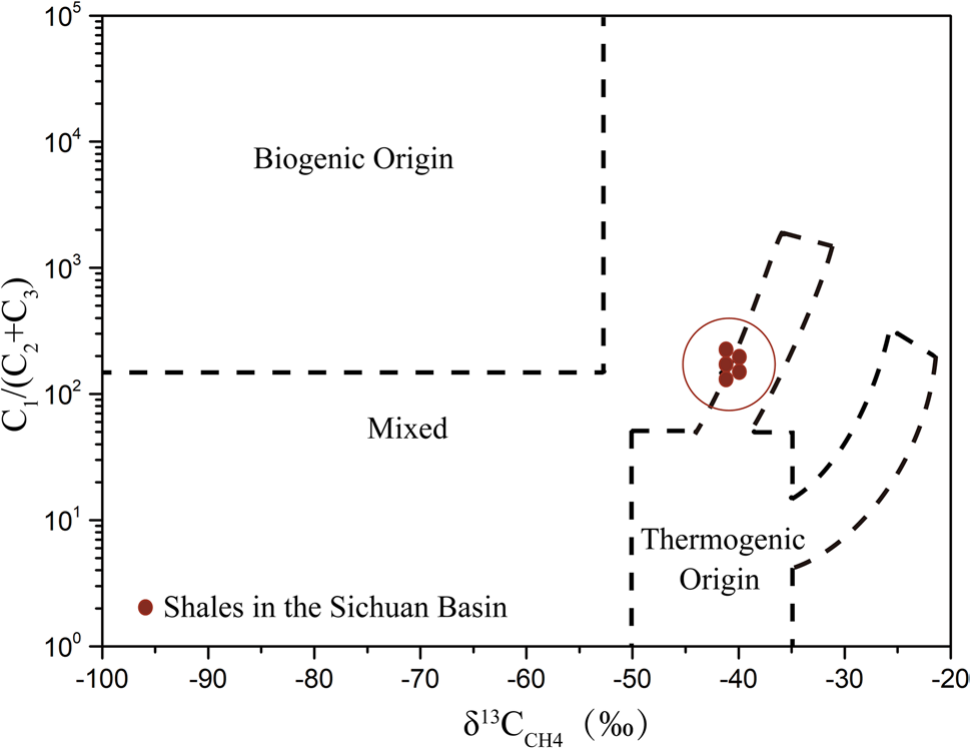
Plot of gas composition C_1_/(C_2_ + C_3_) (methane/(ethane + propane)) versus stable carbon isotope of methane (δ^13^C_CH4_) for shale (red dots) in the Sichuan Basin. The methane origin is categorised according to Whiticar^19^.

Analysis of stable isotopic composition (Fig. 1, Supplementary Table S1 online) indicated that the shale gas in the Sichuan Basin was of thermogenic origin. Stable carbon isotope ratios of CH_4_ (δ^13^C_CH4_) ranged from − 42.5‰ to − 41.3‰, within the theoretically anticipated CH_4_ thermogenic range of − 50‰ to − 20‰^19^. For the stable carbon isotope of CO_2_ (δ^13^C_CO2_), the values ranged from − 21.9‰ to  − 16.8‰, also within the theoretical range of − 30‰ to − 10‰ for thermogenic origin^20^. In addition, the values of (CH_4_/(C_2_H_6_ + C_3_H_8_)) versus δ^13^C_CH4_ were within the fields for thermogenic gas in the plot^19^ (Fig. 1). Compared with the stable isotopic values detected one year ago, both δ^13^C_CH4_ (ANOVA, *p* < 0.001) and δD_CH4_ (ANOVA, *p* < 0.01) had decreased (Supplementary Table S1 online).

### Abundance of taxonomic and functional genes

**Figure 2:**
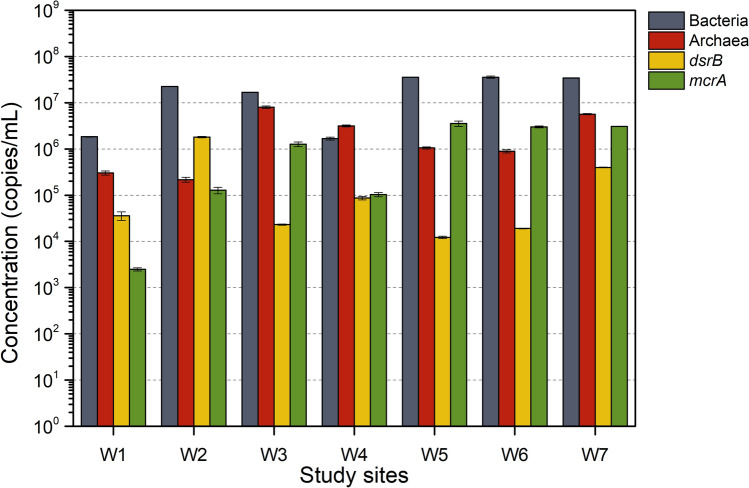
Quantitative PCR (qPCR) results for 16S rRNA genes of bacteria, archaea and function genes of sulfate-reducing prokaryote (*dsrB*) and methanogens (*mcrA*).

Microbial communities (as estimated by 16S rRNA gene copies/mL water), including bacteria and archaea, were quantified (Fig. 2). Assuming an average of 4.19 copies of 16S rRNA gene per bacterium and 1.71 copies of 16S rRNA gene per archaeon genome^21,22^, produced water hosted a microbial community of 3.99 × 10^5^–8.53 × 10^6^ bacterial cells/mL and 1.27 × 10^5^–4.69 × 10^6^ archaeal cells/mL. The number of bacteria in most wells (W1, W2, W5, W6, and W7) was at the same order or one order of magnitude higher than the number of archaea, except for two wells (W3 and W4) where the archaea number was somewhat higher than the bacterial number (Fig. 2).

In all analyzed samples, the detection of marker genes, *mcrA* (averaging 2.48 × 10^3^–3.54 × 10^6^ copies/mL water) and *dsrB* (averaging 1.23 × 10^4^–1.8 × 10^6^ copies/mL water), was indicative for coexistence of methanogens and SRP (Fig. 2). Assuming that per SRP has a single copy of *dsrB* gene^23^ and per methanogen has a single copy of *mcrA*^24^, the average number of SRP and methanogen in produced water was 3.40 × 10^5^ cells/mL and 1.59 × 10^6^ cells/mL, respectively. Given the percentage in the total number of bacteria and archaea, SRP and methanogens accounted for 6.7% and 17.2%, respectively.

### Microbial community composition in produced water

**Figure 3:**
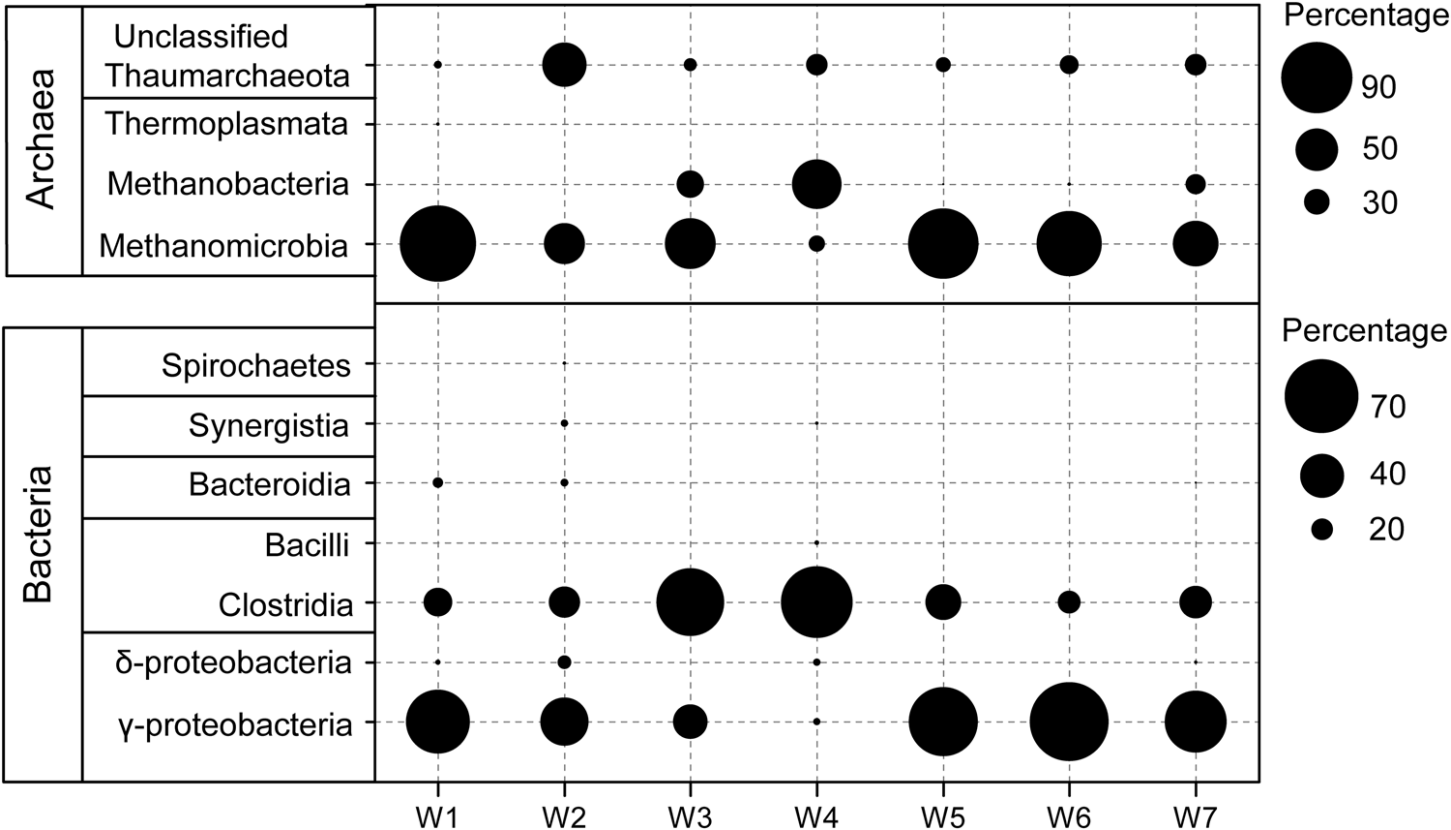
Relative abundance of bacterial and archaeal sequences at class level in produced water from shale gas wells based on 16S rRNA gene sequencing.

**Figure 4:**
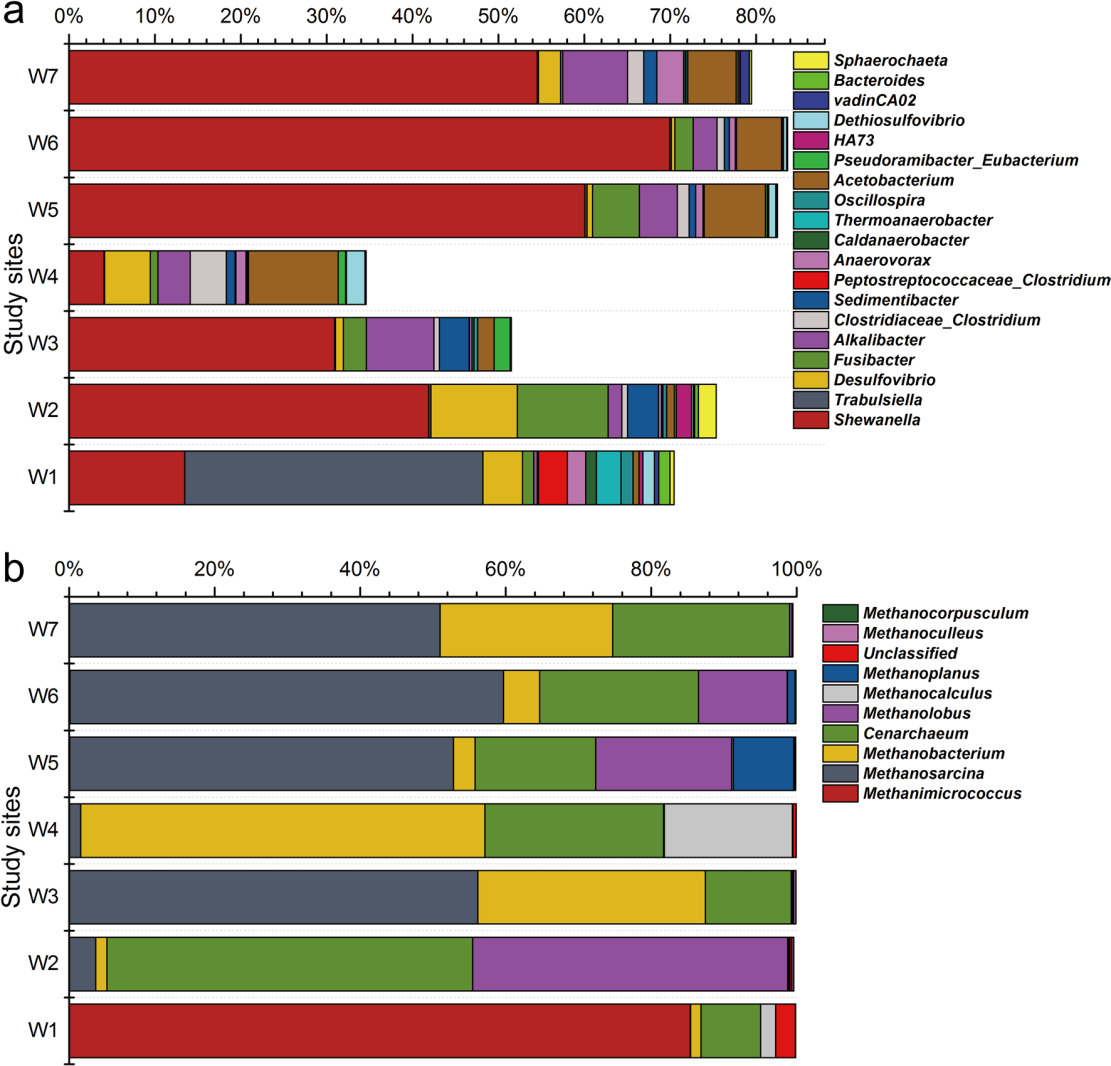
Relative abundance of bacterial and archaeal sequences at genus level in produced water from shale-gas wells based on 16S rRNA gene sequencing.

Microorganisms unique to the deep shale-gas wells were revealed by 16S rRNA gene sequencing. We recovered sequences affiliated to the classes *γ-Proteobacteria* within *Proteobacteria* and *Clostridia* within *Firmicutes* that constituted the two most-abundant fractions of bacterial community (31–70% and 21–63%, respectively, of the total effective bacterial sequences) (Fig. 3). The result that *γ-Proteobacteria* dominated in most samples here, differed from the result of previous study which showed fermentative halotolerant *Clostridia* species were the last “winner” that consisted almost entirely bacterial sequences^25^. At the genus level, *Shewanella* was the most abundant genus (averaging 61%), followed by sulfate-reducing *Desulfovibrio*^26^ (averaging 4%) and thiosulfate-reducing *Fusibacter*^27^ (averaging 3%) given the average abundance of all the samples (Fig. 4a).

For archaea (Figs. 3, 4b), the most dominant phylum was *Euryachaeota* (averaging 77%), followed by unclassified *Thaumarchaeota* (averaging 23%). At the genus level, the most abundant archaeal genus was *Methanosarcina* (averaging 45%), which is a very robust methanogen and generates methane via all three methanogenesis pathways^28,29^, followed by *Methanobacterium* (averaging 17%) performing hydrogenotrophic and methylotrophic methanogenesis^29^, and *Methanimicrococcus* (averaging 12%) performing methylotrophic methanogenesis^30^.

### Functional genes related to methanogenic pathway

**Figure 5:**
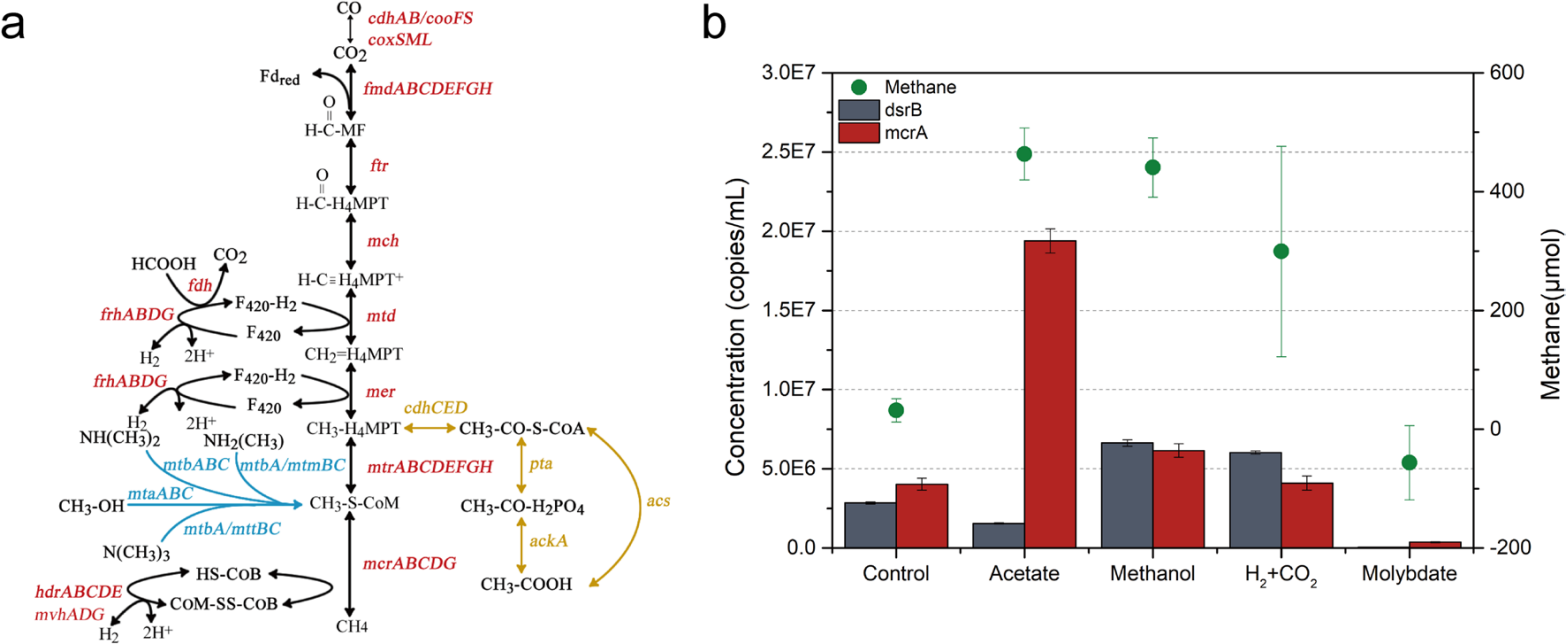
Methanogenic pathways and biogenic methane production potential of deep shale-gas wells after hydraulic fracturing. a. The proposed methanogenetic pathways and related genes detected in shale-gas wells. Genes shown in red colour are involved in the hydrogenotrophic pathway, blue colour in the methylotrophic pathway, yellow color in the acetoclastic pathway. b. Quantification of *dsrB* and *mcrA* genes (bar), and methane generation (green dot) from produced water samples supplemented with deionized water (Control), acetate, methanol, H_2_ + CO_2_, and sodium molybdate (Molybdate) on the day of 110.

The functional pathways were investigated using a KEGG module mapper to examine critical genes in shale-gas metagenomes. As shown in Fig. 5a, all gene homologues that encode enzymes directly responsible for the three complete methanogenesis pathways, namely, hydrogenotrophic, acetoclastic and methylotrophic methanogenesis, were identified. Key genes related to hydrogenotrophic methanogenesis involved *fmd*, *ftr*, *mch* and *mer*. Acetoclastic genes including *ack*, *pta* and *cdh* as well as alternative acetyl-CoA synthetase-encoded gene *acs* were also identified. For methylotrophic methanogenesis, *mtb* and *mta* which encode functional enzymes catalyzing methanol and methylamines to form methane were detected. In addition, abundant energy conserving genes including *hdr*, *ech*, *nah* and *eha* that took part in methane formation were detected here (Supplementary Table S2 online). A detailed description of the three methanogenic pathways and energy conserving genes is presented in Supplementary Information.

### Biogenic methane production tests

Methane production was observed in all the enrichments supplemented with acetate (463 ± 44 μmol), methanol (441 ± 49 μmol), and H_2_ + CO_2_ (299 ± 177 μmol), even the negative control only with distilled water had a small amount of methane emission (32 ± 20 μmol), indicating that the methanogens existed in these deep shale-gas wells were active and could be stimulated to produce methane through all the three methanogenic pathways (Fig. 5b). At the present substrate concentrations in this study, acetate seemed to be the most favorite substrate for methanogens as acetate supplement group produced the highest amount of methane with the largest number of *mcrA* genes (averaging 1.9 × 10^7^ copies/ml) and the lowest number of *dsrB* genes (averaging 1.5 × 10^6^ copies/ml) among all the enrichments till the end of incubation experiments (Supplementary Fig. S1).

### Influence of geochemical factors on methane formation

The geochemical factors were shown in Table 1. The pH values of the produced water ranged from 6.8 to 8.0. Conductivity used for estimating the total ionic content ranged from 20.2 to 83.8. Consistent with our previous reports on produced water from the shales in the Sichuan Basin^10^, Cl^-^, Na^+^, K^+^, Ca^2+^, Ba^2+^ and Mg^2+^ were still the dominant ions. However, the concentration of SO_4_^2-^ and NO_3_^-^ decreased alone with continued gas extraction. It was noteworthy that Cl^-^ concentration here was lower than that in other reported shales including Marcellus^4,31^, Haynesville^11^ and Antrim shales^9,32^ (Cl^-^ > 50,000 mg/L).

**Table 1:** Descriptions of water samples from sampled wells.

Study sites	W1	W2	W3	W4	W5	W6	W7
Well age (month)^a^	10	15	14	14	16	7	7
pH	8.0	7.7	7.7	7.7	6.8	7.6	7.7
DO (mg/L)	0.1	0.5	0.6	0.5	0.4	0.4	0.6
Conductivity(ms/cm)	20.2	54.3	56.3	57.5	83.8	46.6	50.5
DOC (mg/L)	127.8	74.4	46.1	62.9	71.5	60.9	77.9
DIC (mg/L)	111.7	147.5	92.4	123.1	54.9	91.4	137.4
TDN (mg/L)	28.8	36.0	33.8	33.6	40.2	29.9	30.1
B (mg/L)	14.6	12.4	23.4	10.0	10.3	10.6	11.1
Ba (mg/L)	17.2	105.3	315.2	164.2	327.2	78.0	82.7
Ca (mg/L)	76.6	172.8	268.0	141.5	495.0	165.9	163.8
K (mg/L)	101.6	147.1	204.8	130.6	214.0	123	131.7
Li (mg/L)	16.0	22.0	40.0	28.3	37.2	24.1	24.5
Mg (mg/L)	9.2	29.0	33.5	28.7	87.2	27.8	28.0
Na (mg/L)	3388	6382	9876	6764	10,142	5656	5948
Sr (mg/L)	16.0	74.6	106.1	68.4	166.7	60.6	57.6
SO_4_ (mg/L)	11.2	5.5	10.4	4.0	7.3	5.3	5.3
Cl (mg/L)	6864	20,115	19,436	22,749	34,541	18,015	18,805
F (mg/L)	10.6	37.0	14.7	BDL	BDL	22.6	BDL
Br (mg/L)	BDL	BDL	88.5	61.1	BDL	49.1	BDL
NO_3_ (mg/L)	BDL	BDL	24.2	BDL	BDL	BDL	BDL
Total Si (mg/L)^b^	52.5	53.8	56.0	38.9	37.8	51.0	63.6
Total Fe(mg/L)	1.4	47.4	54.4	31.7	16.9	22.9	34.9

*DIC* Dissolved inorganic carbon; *DOC* dissolved organic carbon; *TDN* total dissolved nitrogen; *BDL* below detection limit.

^a^The period from gas production to sampling.

^b^Display as the concentration of SiO_2_.

**Figure 6:**
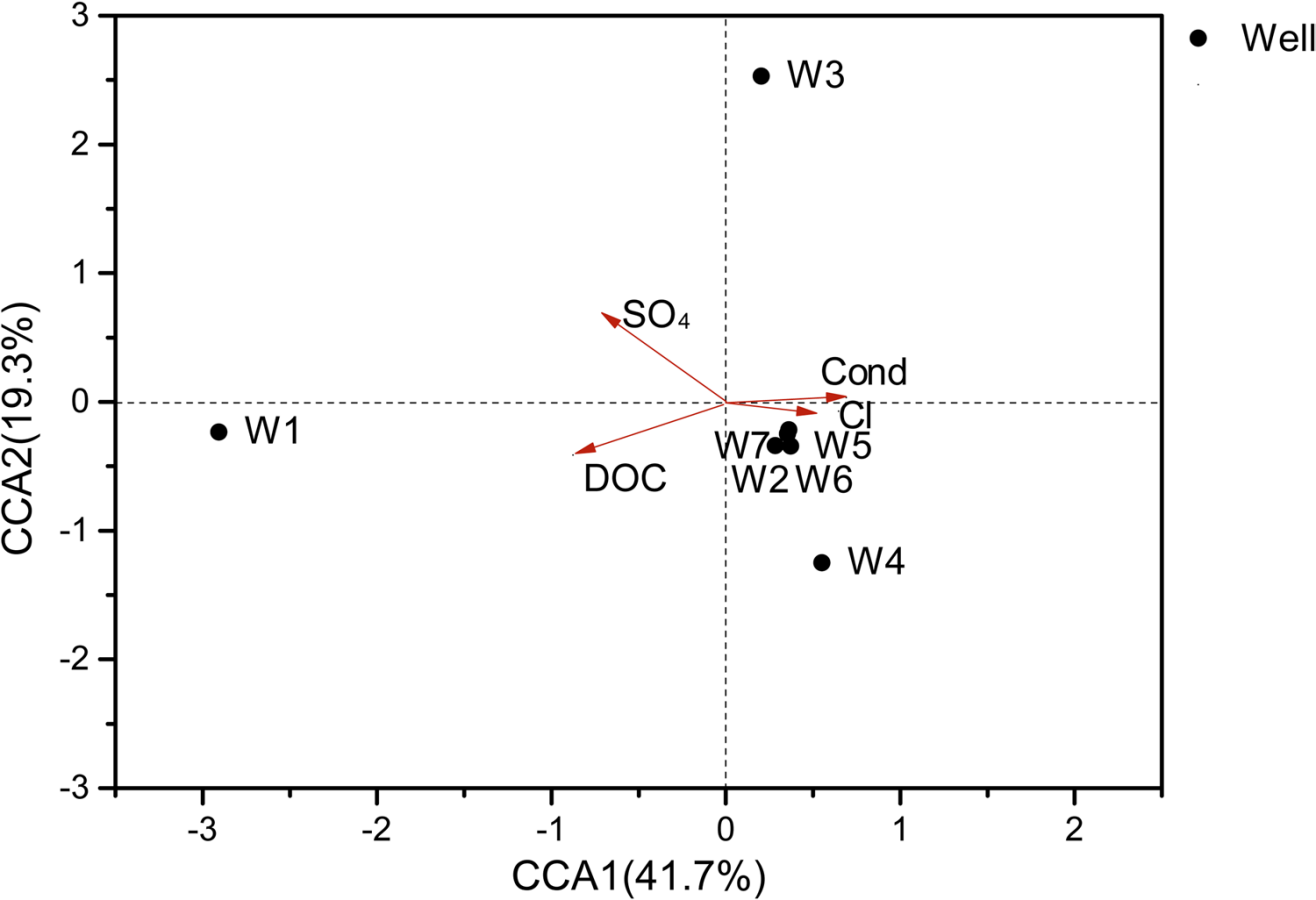
Canonical correlation analysis (CCA) of methane (**a**) and sulfur- (**b**) related microorganisms with geochemical factors of produced water. The four major geochemical factors that influence microbial distribution are displayed in the plot. Abbreviations ''Cond'' indicates ''Conductivity'', ''DOC'' indicates ''Dissolved organic carbon''.

Canonical correlation analysis (CCA) was performed to evaluate the relationships between methanogenic related microbes and geochemical parameters. The total canonical eigenvalue explained by the two axes (CCA1 and CCA2) for microbial distribution related to methane formation was 63%. Four environmental parameters mainly influenced microbial distribution, including DOC, SO_4_^2-^, Cl^-^ and conductivity, are shown in Fig. 6. Specifically, similar to previous findings in other natural environments^33^, SO_4_^2-^ concentrations considerably influenced the distribution of methanogenic microbes (Envfit, r^2^ = 0.54, *P* < 0.1).

**Figure 7:**
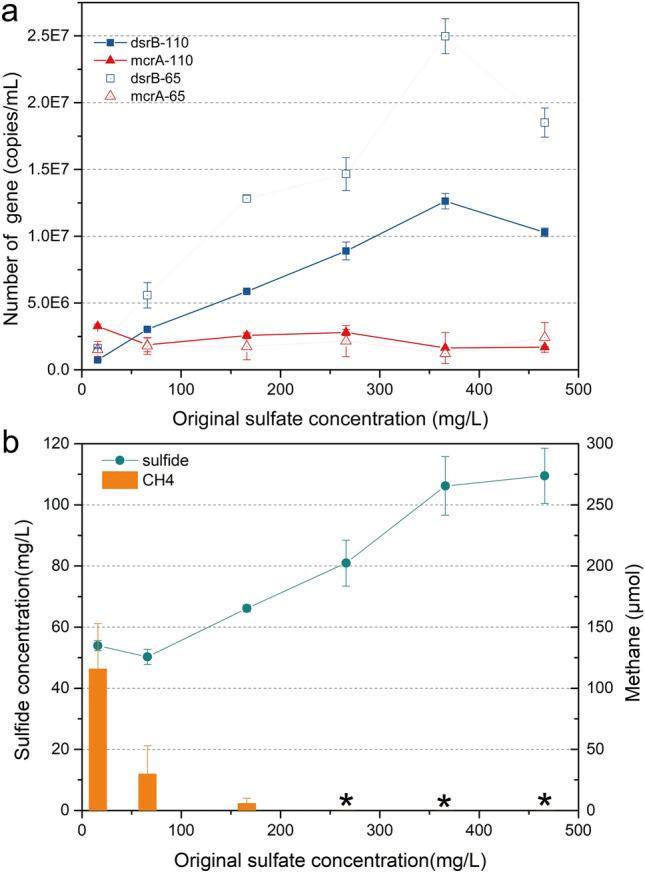
Quantitative PCR (qPCR) of function genes (**a**) and the production of methane and sulfide (**b**) under gradient of sulfate concentration ''*dsrB*-65/ *mcrA*-65′' and ''*dsrB*-110/*mcrA*-110′' indicates the number of these function genes on incubation days 65 and 110, respectively. ''*'' indicates no methane production observed during incubation.

Based on the result of CCA analysis, incubation experiments were designed to further determine the effect of sulfate concentration on methane production. Under low original sulfate concentration (9.0 mg/L) approaching to in situ sulfate concentration in wells, the number of *mcrA* genes and methane production were higher on day 110 of incubation than that on day 65 of incubation (ANOVA, *p* < 0.001). Additionally, the treatment group adding molybdate to inhibit sulfate reduction in methane production tests showed reduced methane production (-56 ± 62 μmol) along with suppressed *dsrB* (averaging 5.7 × 10^4^ copies/ml) and *mcrA* (averaging 3.7 × 10^5^ copies/ml) expression (Fig. 5b), suggesting the cooperation of methanogens and sulfate reducers under in situ sulfate concentration. However, when the original sulfate concentration increased (66–466 mg/L), the number of *mcrA* genes decreased to a low level, while the number of *dsrB* genes increased along with the increasing sulfate concentration (Fig. 7a). This results, combined with the observation of decreased methane production and increased sulfide concentration (Fig. 7b), suggested that methanogenesis was totally or partially inhibited, while sulfate reduction was significantly motivated when sulfate concentration increased far from the in situ concentration.

## Discussion

The chemical composition and stable isotopic ratios of shale gas indicate that the sampled shales in the Sichuan Basin are of thermogenic origin. Despite of this, the value of δ^13^C_CH4_ and δD_CH4_ had a decreased trend along with continued commercial exploration. Once shale gas extraction began, the microbial community in reservoirs changed due to the increased available carbon and energy sources induced by hydrofracturing fluids. Continued microbial activities, especially methanogenic activity, possibly contribute to the observed shifts in the isotopic composition of CH_4_ by generating CH_4_ in conditions that are more consistent with an open system than they are before development^32^.

One of the significant microbial features in present shale-gas wells is the dominance of *Shewanella* sp., which produce sulfide and possibly play important roles in sulfur metabolism and methanogenic activities in present subsurface ecosystem^10^. Members of *Shewanella* are the most diverse respiratory organisms described so far. Approximately 20 organic and inorganic compounds can be respired by *Shewanella* as electron acceptors^34^. On one hand, *Shewanella* produces hydrogen sulfide (H_2_S) when using thiosulfate, sulfite, or elemental sulfur instead of sulfate as electron acceptors, thereby having a deleterious effect on shale gas production^35^. In fact, such sulfidogenic microbes incapable of using sulfate to produce sulfide were reported in previous study on shale microbes, such as *Halanaerobium* species within Firmicutes^25,36^. Because sulfate is the stable and originally dominant form of oxidized sulfur, any anaerobic respiration of sulfur species has to begin with sulfate reduction. Reduction of sulfur and thiosulfate by *Shewanella* would indicate a preceding incomplete oxidation of sulfide produced by SRP by entering oxidants like oxygen (Table 1). One the other hand, *Shewanella* sp. have been shown to utilize, directly or through soluble electronic relays, oxidized metals such as Fe(III), Mn(III and IV) and Cr(IV) as electron acceptors^34,37,38^. Thus the abnormal increase/decrease of *Shewanella* sp. could be proposed to be a biomarker to detect alteration of such special ecosystems in shale-gas wells^39^. Except for *Shewanella* group, *Geobacter* was another dominant metal-reducing genus which was found in other shale-gas wells recently^12^. The roles of metal-reducing process played by *Shewanella* and *Geobacter* in subsurface ecosystems need to be further investigated. What’s more, under anaerobic conditions in shale formations, *Shewanella* and *Geobacter* have a strong preference for lactate as their carbon source and electron donors with excretion of acetate and CO_2_ as end products, which could be subsequently utilized by acetate-utilizing and CO_2_/H_2_ utilizing microbes, especially methanogens^7,40^. Thereby, they may keep syntrophic relationship with methanogens^40^ in the deep-subsurface environments.

Another microbial feature is that SRP, especially *Desulfovibrio* sp., were identified in all collected produced waters samples here (Fig. 4a). Members of *Desulfovibrio* are able to utilize sulfate, sulfite and thiosulfate serving as terminal electron acceptors to ferment lactate, pyruvate, fumarate, succinate and malate to acetate and CO_2_^26^, which could be used by methanogens as substrates to produce methane. Under sulfate limited conditions of the produced water, H_2_ was also the highly possible end-products of *Desulfovibrio*, which is known to participate in interspecies hydrogen transfer with methanogens^41^. Moreover, *Desulfovibrio* is observed capable of conducting extracellular electron transfer directly via outer-membrane cytochromes^42^, and is speculated to participate in methanogenic process via directly transferring electrons which needs further investigation^43^. Since direct interspecies electron transfer (DIET) may be a more effective mechanism for interspecies electron exchange than reduced molecules such as acetate and hydrogen, it has attracted more and more attention recently^44–46^. Some effective strategies for enhancing methane production by stimulating DIET, such as adding granular activated carbon and semi-conductive minerals, have been employed in methanogenic systems^47,48^. This may also imply great application prospects for stimulating methane production in shale systems, which deserves further study.

To date, most archaeal 16S rRNA gene sequences recovered from shale-gas wells belong to the Euryachaeota^4,9,31,49^, which mainly contains methanogens, thermophiles and halophiles^50^. In addition to this commonly observed archaea in shale formations, high abundance of Thaumarchaeota (Fig. 3) was first detected in these deep shale-gas wells. In addition to the methanogens within Euryachaeota, Thaumarchaeota is the only known archaea that takes part in both carbon and nitrogen cycling in natural environment^51^, which is inferred playing important roles in element cycling in subsurface shale ecosystems. Apart from Thaumarchaeota members, methanogens that could get energy via at least one methanogenic pathways, especially *Methanosarcina* sp.^52^, were detected in present field environments. The dominance of *Methanosarcina* with the ability of using acetate is infrequent in other shales: In fact, methyltrophic *Methanohalophilus*^53^ is reported to be the prevalent methanogen in hydraulically fractured shales including Marcellus^4,31^, Haynesville^11^ and Antrim shales^9,32^. The dominance of this halotolerant methanogen is likely due to the higher salinity concentrations (Cl^-^ > 50,000 mg/L) in these shales than that in shales of the Sichuan Basin (averaging 20,000 mg/L) (Table 1)^50^. Together, *Methanosarcina*, a genus not only performs hydrogenotrophic and methyltrophic methanogenesis but also performs acetoclastic methanogenesis, dominated in the deep low-salinity shales in the Sichuan Basin.

Although in deep-subsurface environments, the results of the laboratory methane-production tests prove that methanogens do exist and are alive. Further, when CO_2_/H_2_, acetate, formate and diverse methyl groups are present, methanogens in the produced water form methane through hydrogenotrophic, acetoclastic and methyltrophic methanogenesis. The results of laboratory tests are supported by field observations on functional genes participating in methanogenic pathways. It is noteworthy that although CO is not a common methanogenic growth substrate, the presence of CO dehydrogenase genes *cooSF*/*cdhAB* here (Fig. 5a) suggests that CO can be possibly utilized as an initial carbon source^54,55^. Diverse metabolic capabilities of the methanogens in such deep subsurface give methanogens more chances to survive in the changing environment via alternative methanogenic pathways to get energy rather than only from one single pathway. Among the three methanogenic pathways, acetoclastic methanogenesis might play noticeable roles in contributing to methane production (Fig. 5b and Supplementary Fig. S1). This result is consistent with the identification of high abundance of acetoclastic *Methanosarcina* identified here (Fig. 4b). As hydrogenotrophic and methyltrophic methanogenesis are observed in most reported shales^9,32,56,57^, the present study is the first to prove the existence and importance of acetoclastic methanogenesis in deep shales with an average depth of 4.1 km.

The field results including the identification of co-existence of methanogen and SRP through high-throughput sequencing and the significant influence of sulfate on methanogens via statistical analysis, made us assume that sulfate was a key factor to affect the relationship of SRP and methanogen in this study. Based on this hypothesis, sulfate reduction and methane production process were then assessed by monitoring functional genes and end-products under different sulfate concentrations in laboratory. The results showed that in contrast to decreased methane production at higher sulfate concentrations, methane production was motivated at low sulfate concentration. And when sulfate reduction was inhibited by molybdate at this low sulfate concentration, methanogenic activity was also suppressed (Fig. 5b), which further proves that SRP and methanogens form syntrophic relationship under in situ conditions with low level of sulfate. In agreement with a previous report^58^, methanogens and SRP are found to have a synergistic association in low sulfate media, with SRP like *Desulfovibrio* identified here that able to produce acetate which is then utilized by acetoclastic methanogens to produce methane. The number of in situ methanogens (17.2%) exceeded SRP (6.7%) based on qPCR test also suggests their syntrophic relationship. In contrast, methane production was suppressed while sulfate reduction was promoted under high sulfate concentration. In these high sulfate environments, inhibition of methanogenesis may be attributed to elevated levels of toxic biogenic sulfide^59–61^. For instance, *Methanosarcina* sp. is reported to have optimally growth in the presence of 43.5 mg/L added sulfide, while it is gradually inhibited along with sulfide increasing to 435 mg/L^59^. Overall, our results suggest the syntrophic relationship between methanogens and SRP depended on the low in situ sulfate concentration. Therefore, sulfate concentration is a significant environmental factor which should be taken into account when regenerating methane by methanogenic microbes in shale-gas wells.

### Conclusions

The contribution of biogenic methane is thought to be less important in deeper shales that shallower shales (< 2.5 km) through previous studies^62–64^ due to higher thermally maturity. However, we prove that although with a thermogenic origin for deep shales, the production potential of biomethane in shales in the Sichuan Basin has been underestimated. High abundance of diverse and active methanogens, especially *Marinasarcina*, with the ability of utilizing a wide range of substrates to gain energy through all the three methanogenic pathways, was detected. This metabolic flexibility makes them successfully survive in the deep subsurface environments. Sulfate concentration controls the co-occurrence of microbial sulfate reduction and methanogenesis. We further demonstrate the partner relationship between SRP and methanogens at low level of in situ sulfate concentration. These results give hope that faster regeneration of unconventional natural gas could be achieved in such deep subsurface, by employing possible biotechnologies to optimize the geochemical parameters to stimulate methanogens, such as increasing available substrate concentration for methanogenesis as well as controlling a low level of sulfate concentration in reservoir conditions.

## Methods

### Sample collection

Shale gas (five samples) and produced water (seven samples) from gas wellheads were collected with the help of trained industry technicians from horizontal gas wells in southeastern Sichuan Basin, China, in September 2015. Wells were drilled to a true vertical depth of 2700–4317 m with at least seven months of production. Water samples (3 L for each well) were collected into sterile polypropylene bottles with no headspace to avoid oxygen entering. Bottles were transported on ice to the laboratory once finishing sampling. Water samples were stored at 4 °C and processed within 24 h. Five gas samples (1 L for each well) were collected at the well sites through water displacement into inverted sterile bottles, which were subsequently sealed tightly underwater with butyl rubber stoppers to prevent oxygen entering.

### Chemical analysis of produced water and gas samples

The pH, conductivity and dissolved oxygen (DO) of the produced water were measured immediately on finishing sample collection in the field using a portable detector (Hach Company, Loveland, CO, USA). Samples for measurements of dissolved inorganic carbon (DIC), dissolved organic carbon (DOC), and total dissolved nitrogen (TDN) were filtered through 0.22 μm syringe filters (EMD Millipore, MA, USA) before detection using Analytikjena multi N/C 3100 TOC/TNb Analyzer (Analytikjena, Jena, Germany). Cations and anions were measured according to Zhang et al.^10^, by inductively coupled plasma optical emission spectroscopy (ICP-OES) (PerkinElmer, Waltham, MA, USA) and Dionex inductively coupled plasma 2100 (ICP-2100) ion chromatogram (Dionex, Sunnyvale, CA, USA), respectively.

The chemical composition of shale gas was determined by Agilent 7890A gas chromatograph (Agilent Technologies, Santa Clara, USA) equipped with a HayeSep Q packing column and a thermal conductivity detector using He as the carrier gas. The stable isotopic ratios of CO_2_ and CH_4_ were measured by an online gas extraction system, Trace GC Ultra (Thermo Electron Corporation, TX, USA), a combustion furnace, and a ThermoQuest Delta^plus^XL isotope ratio mass spectrometer (Thermo Finnigan).

### DNA extraction and sequencing

The biomass in water samples (1 L) was concentrated onto 0.22 μm cellulose-ester filtering membranes (EMD Millipore, MA, USA). Genomic DNA was extracted using DNeasy PowerSoil Kit (QIAGEN, Hilden, Germany) according to the manufacturer’s instructions after membrane shearing. 1% agarose gel electrophoresis was used to approximately check the purity and integrity of extracted DNA. The DNA concentration was examined using NanoDrop One Spectrophotometer (NanoDrop Technologies, Wilmington, DE, USA). Triplicate extracted DNA in the same volume from the three replicates was mixed into the final DNA which were then stored at -20 ℃ before sequencing. Genomic DNA of all the seven produced water samples were conducted 16S rRNA gene sequencing, two of which extracted from water samples (W5 and W6) in two wells with different production period were picked to conduct metagenomic sequencing.

For 16S rRNA gene sequencing, bacterial V4–V5 region and archaeal V4 region of the 16S rRNA gene were amplified to generate PCR libraries. Primer sets and PCR conditions for bacterial and archaeal amplification are presented in Supplementary Information. After quantification with Qubit, the PCR libraries were sequenced on the Illumina HiSeq PE250 platform.

For metagenomic sequencing of total DNA, the construction of sequencing libraries and the determination of the size distribution and concentration of purified products in libraries were performed according to Zhang et al^65^. The sequencing of prepared libraries was performed on the Illumina HiSeq PE250 platform after generating clustered index-coded samples.

### Downstream processing of 16S rRNA gene sequencing and metagenomic sequencing data

For 16S rRNA gene sequencing, raw paired-end reads were assigned to each sample according to its unique barcodes. After cutting of the barcodes and primers, paired-end reads were merged, and controlled quality. Then chimeras were detected and removed, and the effective tags were obtained to cluster into operational taxonomic units (OTUs) at 97% sequence similarity. Next, representative sequences were assigned into taxonomy compared with the GreenGene Database^66^. The sequence data was normalized by extracting the least sequences of bacteria and archaea in each sample for alpha and beta diversity. Alpha diversity indices, including ACE and Chao indicating species richness, Shannon and Simpson indicating diversity, and Good’s coverage at 3% dissimilarity cut-off were calculated with QIIME. Statistical analyses were conducted to value the effects of individual geochemical factors on methanogenic microbes using Envfit in the Vegan package in R software (Version 3.2.0)^67^. The top four major geochemical factors influencing microbial distribution were displayed and combined with sequencing data to perform CCA. Detailed quality results and rarefaction curves (Supplementary Fig. S2 online) of the 16S rRNA gene sequencing data (Supplementary Table S4 online) are shown in Supplementary Information.

For metagenomic sequencing, all sequencing reads (raw data) were subjected to readfq (https://github.com/billzt/readfq) for quality control. The quality-controlled metagenomics reads (clean data) were assembled based on multiple k-mer method (k-mer size values of 49, 55 and 59) using MEGAHIT software (Version 1.0.4-beta)^68^. The obtained scaffolds, with maximum N50 assembled by contigs, were picked and fragmented to scaftigs^69^. Additionally, clean data was mapped to scaftigs of each sample in order to obtain unassembled reads which were assembled and fragmented to scaftigs again. All the scaftigs (≥ 500 bp) were subjected to MetaGeneMark software (Version 2.10) for Open Reading Frame (ORF) prediction^70^. Clean data were aligned to the predicted gene catalogues by Bowtie software (Version 2.24) and the genes with aligned reads number ≤ 2 were removed, which produced the unique genes used in the following process^71^. For functional analyses, the unique genes were blasted against Kyoto Encyclopedia of Genes and Genomes (KEGG) database (Version 2018–01)^72^. The abundance of annotated functional genes was calculated by summing the relative abundance of normalized corresponding unique genes. Detailed quality results and the features of the metagenomics sequencing data (Supplementary Table S5 online) are shown in Supplementary Information.

### Enumeration of gene abundance via quantitative PCR

Quantitative PCR (qPCR) was used to determine the abundance of bacterial and archaeal 16S rRNA genes. In addition, we used qPCR to enumerate the (A) dissimilatory sulfite reductase (*dsrB*) genes using primers (Supplementary Table S3 online) that target both SRP^73^; (B) methyl-coenzyme reductase (*mcrA*) genes using primers (Supplementary Table S3 online) that target methanogenic archaea^74^. Methanogen-specific primers used to detect *mcrA* were able to evaluate all five proposed phylogenetic orders of archaea; therefore, the quantification results reflect the number of the majority of known methanogens^75^. As the primer pair selected to enumerate the marker genes *dsrB* did not target all sulfate-reducing microorganisms, the results only represent the number of sulfate-reducing bacteria (SRB) with low G + C Gram positive, SRB belong to δ-proteobacteria and Nitrospira division, and sulfate-reducing archaea (SRA)^73^. Quantification was performed in triplicate on DNA extracts using the ABI 7500 Sequence Detection System (Applied Biosystems, Foster City, CA, USA). Standard curves for bacteria, archaea, SRP and methanogens were generated from tenfold serial dilutions of plasmids containing 16S rRNA sequences or functional genes of *Desulfosporosinus* sp. (KC215425), *Methanosarcina* sp. (KC215420), *Desulfovibrio* sp. (DSM 642), and *Methanosarcina* sp. (KC244184) from clone libraries. Negative controls without DNA templates were performed for each qPCR assay, giving null or negligible values of copies. The detailed description about reaction conditions, primers, and standard curves of qPCR (Supplementary Fig. S3) are shown in Supplementary Information.

### Incubation experiments for methane production and effect of sulfate

Bottle experiments were conducted by mixing 2 volumes of produced water (mixture of an equal volume of water from all the wells) with 1 volume of anaerobic medium, amended with methanogenic substrates in 100 mL glass bottles. The bottles were sealed with butyl rubber stoppers and an open-hole screw cap. The anaerobic medium was prepared as previously described^75^ in an anaerobic glove box (Xinmiao YQX-11, Shanghai, China). Given that in situ methane could be utilized by microbes^76^ and therefore possibly influence methane production in the original reservoir environment, methane was initially added in the headspace of all the bottles at a ratio of 3 volumes CH_4_ to 2 volumes N_2_ (V/V; 1×10^5^ Pa) in most bottles (except the bottles adding H_2_ + CO_2_). To determine the methane production potential, available methanogenic substrates were provided with acetate (50 mM), methanol (50 mM), H_2_ + CO_2_ (CH_4_/ H_2_ + CO_2_/ N_2_, 3/1/2, V/V/V; 1 ×10^5^ Pa), or sodium molybdate (28 mM), a specific inhibitor of sulfate reduction^77^. In addition, control group without methanogenic substrate but the same amount of deionized water was set up. To examine the effects of sulfate on methanogenesis, treatments under varied sulfate concentrations ranging from 9 mg/L to 466 mg/L were set up. Cultivations were performed in triplicate. All bottles were kept in the dark at 37 °C for 110 days.

Methane production in the headspace was measured once two months using Agilent 7890A gas chromatograph (Agilent Technologies, Santa Clara, USA) equipped with a flame ionization detector, and was converted to micromoles (μmol) according to the ideal gas law, with standard pressures and temperatures. Samples for sulfide analysis on day 110 were fixed firstly with zinc acetate solution for preservation as zinc sulfide. Dissolved sulfide concentrations were subsequently determined by gas-phase molecular absorption spectrometry AJ-3000 plus (Shanghai ANJIE CO.LTD, Shanghai, China)^78^. Every two months, 1 mL liquid was transferred from the anaerobic bottles using a sterile syringe to prepare DNA extraction for qPCR of functional genes (*mcrA* and *dsrB*).

## Supplementary information

Supplementary file1

## Data Availability

Sequencing data were deposited in the NCBI sequence read archive under Bioproject PRJNA287481. The 16S rRNA gene sequencing data can be accessed from Biosample numbers SAMN06216575 to SAMN06216581. Raw sequence data of metagenome were assigned accession number SAMN06347163 and SAMN06347164.
