# Supplementary material for: Regeneration of unconventional natural gas by methanogens co-existing with sulfate-reducing prokaryotes in deep shale wells in China
Source: Sci Rep. 2020 Sep 29;10:16042. doi: 10.1038/s41598-020-73010-6 (PMC7525477; doi:10.1038/s41598-020-73010-6)
Supplement: Supplementary file 1 — Supplementary file1 [file 41598_2020_73010_MOESM1_ESM.docx]

***Supplementary Information***

**Regeneration of Unconventional Natural Gas by Methanogens Co-existing with Sulfate-reducing Prokaryotes in Deep Shale Wells in China**

Yimeng Zhang ^1, 2, 3^, Zhisheng Yu ^1*^, Yiming Zhang ^4^, Hongxun Zhang ^1^

^1^ College of Resources and Environment, University of Chinese Academy of Sciences, Beijing 100049, P.R. China

^2^ Institute of Oceanology, Chinese Academy of Sciences, Qingdao 266071, P.R. China

^3^ Open Studio for Marine Corrosion and Protection, Pilot National Laboratory for Marine Science and Technology (Qingdao), No.1 Wenhai Road, Qingdao 266237, China

^4^ Beijing Municipal Ecological Environment Bureau, Beijing 100048, P.R. China

^*^ Corresponding Author. Tel: +86 10 88256057; fax: +86 10 88256057; E-mail: [yuzs@ucas.ac.cn](mailto:yuzs@ucas.ac.cn); College of Resources and Environment, University of Chinese Academy of Sciences, 19 A Yuquan Road, Beijing 100049, P.R. China

**DNA extraction and sequencing**

The PCR reactions were carried out on a GeneAmp 9700 PCR system (Applied Biosystems, Foster City, CA, USA) with the following conditions: 95 °C for 2 min; followed by 30 cycles of 95 °C for 30 s, 55 °C for 30 s, and 72 °C for 60 s; and a final extension at 72 °C for 5 min. Each 20 μL reaction mixture included 5×FastPfu Buffer, 2 units of FastPfu Polymerase (Stratagene, USA), 250 mM dNTP mix, 0.1 μM each primer, and 1-5 ng template DNA.

**Enumeration of gene abundance via quantitative PCR**

Template DNA (1 μL) was used in a reaction mixture of 25 μL containing 12.5 μL of 2× SYBR Green mix (Fermentas, USA), 1 μL of each primer (10 nM), 0.3 μL bovine serum albumin (BSA; 10 mg/mL; New England Biolabs, MA, USA), and 9.2 μL ddH_2_O. The PCR was initiated at 95 °C for 10 min, followed by 40 cycles of denaturation for 30 s at 95 °C, the annealing temperature (Table S1) for 30 s, and elongation for 30 s at 72 °C. Fluorescence signals were collected at 72 °C during the elongation step. Melting curve and gel electrophoresis analyses were performed to confirm that the amplified products were of the appropriate size. The standard curves were shown in Fig. S1.

**Functional genes related to methanogenic pathway**

For hydrogenotrophic pathway (Fig. 5a), first, formylmethanofuran dehydrogenase (encoded by *fmdABCDEFGH*) catalyzes the formation of formylmethanofuran (formyl-MF) from methanofuran (MF) and CO_2_ with reducing ferredoxin (Fd). The formyl group is next transferred to tetrahydromethanopterin (H_4_MPT) by formylmethanofuran transferase (ecoded by *ftr*) and stepwise reduced to methyl-H_4_MPT. The intermediate reactions are catalyzed by methenyl-H_4_MPT cyclohydrolase (encoded by *mch*), methylene-H_4_MPT dehydrogenase (encoded by *mtd*) and methylene-H4MPT reductase (encoded by *mer*). H_2_ is oxidized by F_420_-reducing hydrogenase (encoded by *frh*) to provide electrons for the last two intermediate reactions. The next reaction is catalyzed by H_4_MPT mythyltransferase (encoded by *mtr*), a membrane-bound eight-unit complex that not only directs the synthesis of methyl-S-CoM, but also generates a sodium ion gradient to drive various energy-requiring reactions as proposed previously in hydrogenotrophic and acetoclastic methanogenesis pathway^1^.

For acetoclastic methanogenesis (Fig. 5a), acetate kinase (encoded by *ack*A) first catalyzes acetate to produce acetylphosphate which then is catalyzed by phosphotransacetylase (encoded by *pta*) to form acetyl-CoA. The acetyl-CoA is cleaverd at the C-C and C-S bonds to form methy-H4MPT by the bi-functional CO dehydrogenase/acetyl-CoA synthase (encoded by *cdh*)^2^. An alternative enzyme for catalyze acetate to acetyl-CoA directly is acetyl-CoA synthetase (encoded by *acs*) in some acetate-grown methanogens that lack of the above two enzymes^3^.

The terminal reaction (Fig. 5a) to produce CH_4_ as well as CoM and CoB disulfide (CoM-SS-CoB) is catalyzed by methypriul-CoM reductase complex (MCR) (encoded by *mcrABCDG*), the most important enzyme during all methanogenic pathways in methanogens. Heterodisulfide reductase (encoded by *hdrABC/DE*) reduced the heterodisulfide CoM-SS-CoB with H_2_ to CoM and CoB, along with the last electron transport reaction to generate a proton gradient that drives ATP synthesis. Some methanogens contains these two distinct classes of heterodisulfide reductase complex, such as *Methanosarcina mazei*, *M. barkeri* and *M. acetivorans*. However, the roles of *hdrABC* and *hdrDE* may be different in methanogenesis. For *M. acetivorans*, *hdrED* genes are required for viability under growth conditions with acetate and methylated compounds, while *hdrABC* genes appears to be specifically involved in methylotrophic methanogenesis^4^.

Abundant energy conserving genes taking part in methane formation were detected here (Fig. 5a, Table S5). For methanogens, H4MPT methyltransferase, encoded by *mtr* genes, is an important membrane-bound eight-unit complex. It not only directs the synthesis of methyl-S-CoM, but also generates a sodium ion gradient to drive various energy-requiring reactions as proposed previously in hydrogenotrophic and acetoclastic methanogenesis pathways^1^. We detected key energy-conserving genes in produced-water metagenomes that encode both cytoplasmic (*hdrABC*) and membrane-associated (*hdrDE*) heterodisulfide reductase (Hdr). These encoded enzymes catalyze the last methane-generation reaction, along with the last electron-transport reaction, to generate a proton gradient that drives ATP synthesis. We also tested genes (*echABCDEF*) that encode an important energy-converting [NiFe]-hydrogenase (Ech). Ech is a nickel-iron-sulfur protein, in which the EchE subunit harbours the active-site [NiFe] centre, and is regarded as proton-translocating, as the conversion of CO to CO_2_ and H_2_ in *M. barkeri*^5,6^. In addition, *nhaABCR* genes were found to encode the Na^+^/H^+^ antiporter Nha, an enzyme assumed to be involved in the build-up of the proton motive force^7^. Additionally, we identified [NiFe]-hydrogenase MvhADG (encoded by *mvhADG*), an enzyme coupled with HdrABC to direct the first and last step in hydrogenotrophic methanogens^8^. However, given the dominance of *Methanosarsina* species in microbial communities, the absence of another important membrane-bound protein, [NiFe]-hydrogenase VhoACG, which couples with HdrED to reduce CoM-SS-CoB with H_2_ in almost all Methanosarsinales members^7^, was unexpected. Thus, we propose that this reaction was likely catalyzed by the Rhodobacter nitrogen fixation (Rnf) complex^9^.

Table S1. Chemical composition and stable isotope analysis of shale gas.

| Study sites | (‰ PDB) | | | Major gas composition (mole %) | | |
| --- | --- | --- | --- | --- | --- | --- |
|  | δ^13^C_CH4_ | δ^13^C_CO2_ | δD_CH4_ | CH_4_ | C_2_H_6_ | CO_2_ |
|  | 2014/2015 |  | 2014/2015 |  |  |  |
| W1 | -31.3/-41.3 | -21.9 | -147.5/-175.0 | 92.11 | 0.55 | 3.50 |
| W2 | -29.5/-41.3 | -19.8 | -148.5/-171.0 | 91.23 | 0.64 | 4.00 |
| W3 | -30.8/-41.3 | -20.8 | -146.6/-173.0 | 93.30 | 0.43 | 4.91 |
| W4 | -30.4/-41.6 | -16.8 | -147.4/-184.5 | 94.04 | 0.67 | 3.83 |
| W5 | -31.5/-42.5 | -17.2 | -147.5/-195.5 | 90.57 | 0.56 | 3.09 |

Table S2. Identification of energy conserving genes in methane formation based on KEGG annotation.

| Enzyme name | Gene | Number of unigenes | |
| --- | --- | --- | --- |
|  |  | W5 | W6 |
| heterodisulfide reductase | *hdrA* | 4260 | 3737 |
|  | *hdrB* | 5322 | 4088 |
|  | *hdrC* | 5101 | 3889 |
|  | *hdrD* | 1950 | 1857 |
|  | *hdrE* | 1686 | 1648 |
| tetrahydromethanopterin S-methyltransferase | *mtrA* | 9017 | 7781 |
|  | *mtrB* | 2480 | 1990 |
|  | *mtrC* | 2919 | 2381 |
|  | *mtrD* | 2867 | 2174 |
|  | *mtrE* | 2349 | 2074 |
|  | *mtrF* | 1510 | 1312 |
|  | *mtrG* | 995 | 971 |
|  | *mtrH* | 2779 | 2158 |
| ech hydrogenase | *echA* | 1607 | 1606 |
|  | *echB* | 1490 | 1532 |
|  | *echC* | 44 | 23 |
|  | *echD* | 1644 | 1565 |
|  | *echE* | 1532 | 1414 |
|  | *echF* | 1178 | 1200 |
| Na+:H+ antiporter | *nhaA* | 1465 | 2018 |
|  | *nhaB* | 1601 | 2316 |
|  | *nhaC* | 618 | 396 |
| transcriptional activator of nhaA | *nhaR* | 1771 | 2379 |
| F420-non-reducing hydrogenase large subunit | *mvhA,vhuA,vhcA* | 155 | 63 |
| F420-non-reducing hydrogenase iron-sulfur subunit | *mvhD,vhuD,vhcD* | 136 | 51 |
| F420-non-reducing hydrogenase small subunit | *mvhG,vhuG,vhcG* | 375 | 163 |
| energy-converting hydrogenase A | *ehaB* | 296 | 88 |
|  | *ehaC* | 51 | 16 |
|  | *ehaD* | 169 | 51 |
|  | *ehaE* | 107 | 21 |
|  | *ehaF* | 266 | 57 |
|  | *ehaG* | 153 | 35 |
|  | *ehaH* | 289 | 90 |
|  | *ehaJ* | 200 | 51 |
|  | *ehaN* | 283 | 57 |
|  | *ehaO* | 321 | 78 |
|  | *ehaP* | 290 | 91 |
| energy-converting hydrogenase B | *ehaQ* | 407 | 84 |
| V/A-type H+-transporting ATPase subunit G/H | *ATPVG,ahaH,atpH* | 2270 | 1316 |
| electron transport complex protein | *rnfG* | 2808 | 2811 |
|  | *rnfE* | 2634 | 2848 |
|  | *rnfD* | 2627 | 2790 |
|  | *rnfC* | 1722 | 1831 |
|  | *rnfB* | 1418 | 1662 |
|  | *rnfA* | 1959 | 2444 |

Table S3. Primers used for qPCR of 16S rRNA and functional genes.

| Target group | Target  gene | Primer set (5’ to 3’) | T^b^  (^O^C) | Reference |
| --- | --- | --- | --- | --- |
| Archaea | 16S rRNA gene | 519F: GTGCCAGCMGCCGCGG  915R: GTGCTCCCCCGCCAATTCCT | 60 | 10 |
| Bacteria | 16S rRNA gene | 338F: ACTCCTACGGGAGGCAGC  518R: ATTACCGCGGCTGCTGG | 60 | 10 |
|  |  | AR736r: CCTCAGACCCGTTCTCCTA |  |  |
| Methanogen | mcrA | MLf:  GGTGGTGTMGGATTCACACARTAYGCWACAGC  MLr: TTCATTGCRTAGTTWGGRTAGTT | 58 | 11 |
| SRB | dsrB | DSRp2060F: CAACATCGTYCAYACCCAGGG  DSR-4R: GTGTAGCAGTTACCGCA | 55 | 12 |

^a^ These primers consist of a mixture of each taxonomic group targeted primers at an equal amount (mol).

^b^ Annealing temperature.

Table S4. Diversity estimates of bacteria and archaea detected by 16S rRNA gene sequencing from produced water.

| Well | Reads^a^ | OTU_0.03_^b^ | Chao 1 | ACE | Coverage | Shannon | Simpson |
| --- | --- | --- | --- | --- | --- | --- | --- |
| Bacteria | | | | | | | |
| W1 | 37 298 | 98 | 97.143 | 97.318 | 1.000 | 3.634 | 0.840 |
| W2 | 59 213 | 129 | 170.600 | 141.199 | 0.999 | 3.993 | 0.883 |
| W3 | 45 908 | 113 | 121.545 | 121.380 | 0.999 | 3.411 | 0.825 |
| W4 | 49 355 | 124 | 120.118 | 122.576 | 1.000 | 4.060 | 0.892 |
| W5 | 44 534 | 118 | 110.750 | 112.690 | 1.000 | 2.724 | 0.634 |
| W6 | 54 075 | 120 | 112.571 | 114.892 | 1.000 | 2.194 | 0.514 |
| W7 | 53 035 | 126 | 119.056 | 123.495 | 1.000 | 3.185 | 0.709 |
| Archaea | | | | | | | |
| W1 | 45 956 | 56 | 53.250 | 53.956 | 1.000 | 1.215 | 0.269 |
| W2 | 44 994 | 113 | 170.000 | 128.653 | 1.000 | 3.324 | 0.780 |
| W3 | 47 072 | 85 | 82.091 | 84.115 | 1.000 | 1.824 | 0.587 |
| W4 | 48 494 | 87 | 90.143 | 89.041 | 1.000 | 2.465 | 0.654 |
| W5 | 50 837 | 106 | 102.545 | 104.135 | 1.000 | 2.580 | 0.676 |
| W6 | 46 321 | 100 | 106.667 | 106.119 | 1.000 | 2.406 | 0.619 |
| W7 | 52 915 | 110 | 103.667 | 104.512 | 1.000 | 2.630 | 0.679 |

^a^ Effective reads past quality-control and chimera-remove

^b^ Diversity and richness estimates are based on ≥97% sequence identity

Table S5. Features of the metagenome data generated by Illumina HiSeq sequencing of two produced water samples.

| Study sites | W5 | W6 | MIX^a^ |
| --- | --- | --- | --- |
| *Quality control data* | | | |
| Number of raw reads post- QC | 6471230 | 6450400 | - |
| GC content of clean data ^b^ (%) | 45.98 | 47.75 | - |
| *Assembly of clean data* | | | |
| Total length of scaftigs (bp) | 50 312 969 | 44 033 039 | 11 572 347 |
| Number of scaftigs | 19 527 | 21 724 | 4 357 |
| Average scaftig length (bp) | 2577 | 2027 | 2655 |
| Largest scaftig length(bp) | 383 568 | 212 597 | 237 018 |
| N50 length (bp) | 6211 | 4084 | 22 531 |
| *Prediction of sacftigs* | | | |
| Number of predicted ORFs | 58 035 | 53 495 | 13 023 |
| Total length of predicted ORFs (bp) | 43 730 | 38 520 | 9 660 |
| Average ORFs length (bp) | 753.45 | 720.18 | 741.92 |
| Number of unique genes | 57170 | 56941 | - |

Abbreviation: QC, Quality Control; ORF: Open Reading Frame.

^a^Unassembled metagenomic reads of all the samples were mixed into one sample when assembly and prediction.

^b^Quality-controlled metagenomics reads


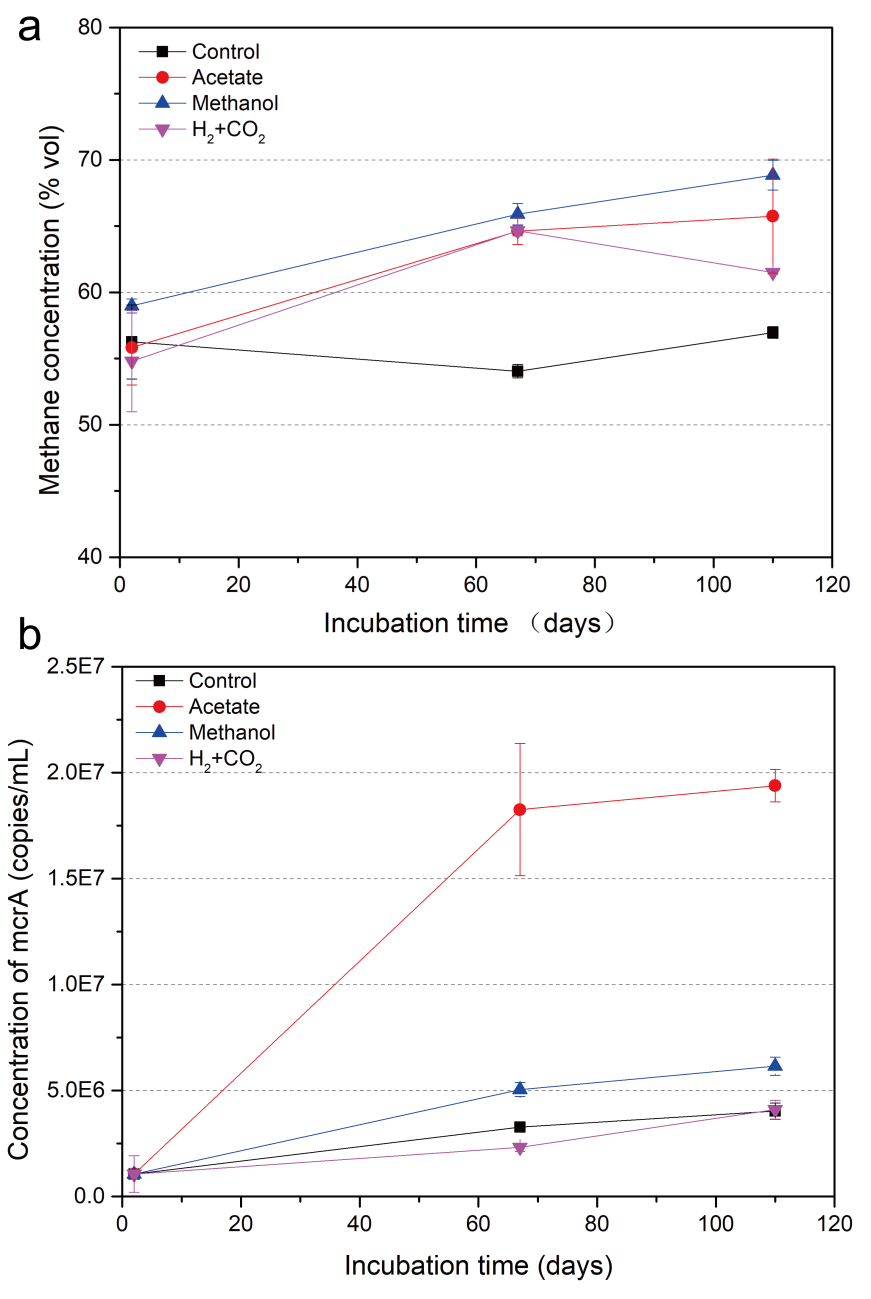


Figure S1 Methane production (a) and *mcrA* genes (b) from the produced water samples supplemented with sterilized water (Control), acetate, methanol, and H_2_+CO_2_. Values are the mean of three replicates indicating the methane volume concentration (a) and the number of *mcrA* genes in the bottles.


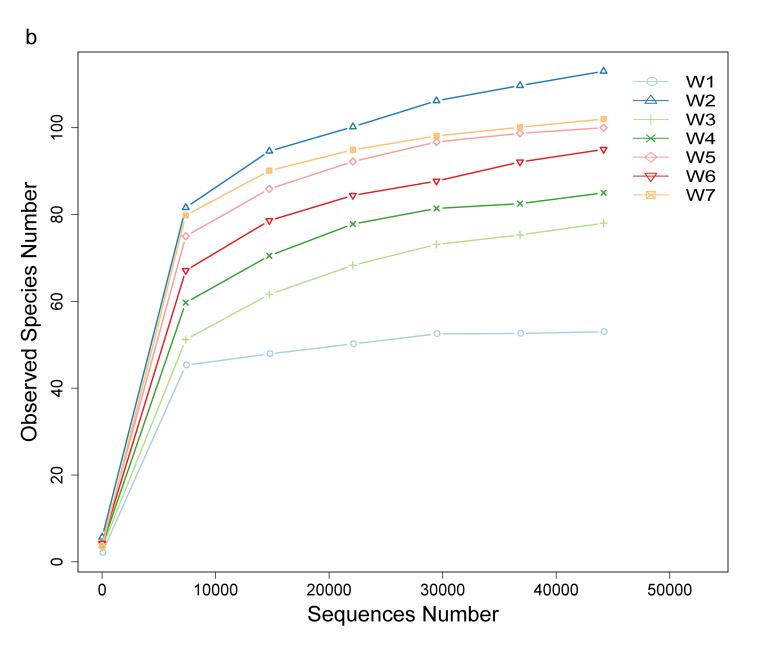

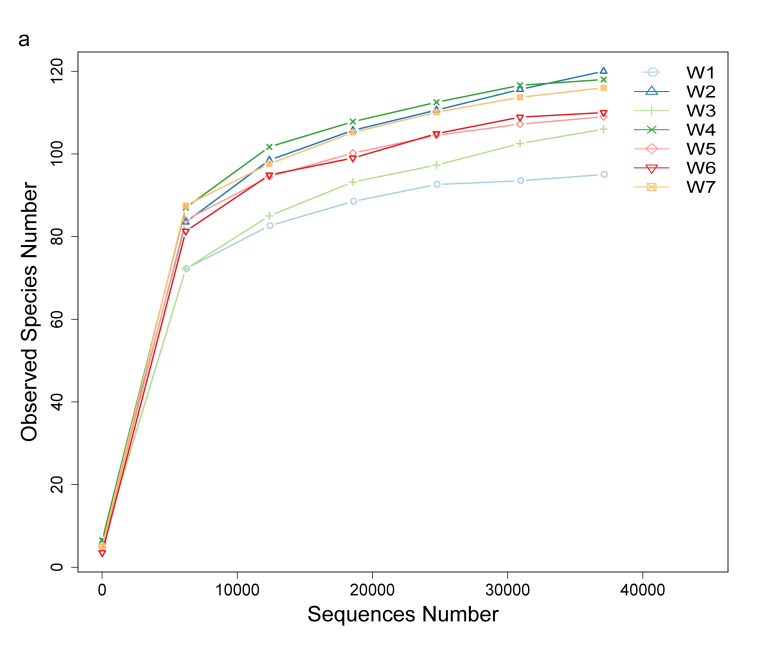


Figure S2 Rarefaction curves of operational taxonomic units (OTUs) for bacteria (a) and archaea (b) of seven produced water samples.











Figure S3 Standard curves of qPCR for bacteria (a), archaea (b), *mcrA* (c) and *dsrB* (d).

**References**

1. Harms, U., Weiss, D.S., Gartner, P., Linder, D. & Thauer, R.K. The energy conserving N-5-methyltetrahydromethanoptetin-coenzyme-M methyltransferase complex from *Methanobacterium thermoautotrophicumis* composed of 8 different subunits. *Eur. J. Biochem.* **228**:640-648 (1995).

2. Fischer, R. & Thauer, R.K. Ferredoxin-dependent methane formation from acetate in cell-extracts of *Methanosarcina barkeri* (strain MS). *FEBS. Lett.* **269**:368-372 (1990).

3. Jetten, M.S.M., Stams, A.J.M., Zehnder, A.J.B. Isolation and characterization of acetyl-coenzyme-A synthetase from *Methanothrix soehngenii*. *J. Bacteriol.* **171**:5430-5435 (1989).

4. Buan, N.R. & Metcalf, W.W. Methanogenesis by *Methanosarcina acetivorans* involves two structurally and functionally distinct classes of heterodisulfide reductase. *Mol Microbiol.* **75**:843-853 (2010).

5. Meuer, J., Kuettner, H.C., Zhang, J.K., Hedderich. R. & Metcalf, W.W. Genetic analysis of the archaeon *Methanosarcina barkeri* Fusaro reveals a central role for Ech hydrogenase and ferredoxin in methanogenesis and carbon fixation. *Proc. Natl. Acad. Sci. U. S. A.* **99**:5632-5637 (2002).

6. Stojanowic, A. & Hedderich, R. CO_2_ reduction to the level of formylmethanofuran in *Methanosarcina barkeri* is non-energy driven when CO is the electron donor. *FEMS Microbiol. Lett.* **235**:163-167 (2004).

7. Thauer, R.K., Kaster, A.K., Seedorf, H., Buckel, W. & Hedderich, R. Methanogenic archaea: ecologically relevant differences in energy conservation. *Nat. Rev. Microbiol.* **6**:579-591 (2008).

8. Kaster, A.K., Moll, J., Parey, K. & Thauer, R.K. Coupling of ferredoxin and heterodisulfide reduction via electron bifurcation in hydrogenotrophic methanogenic archaea. *Proc. Natl. Acad. Sci. U. S. A.* **108**:2981-2986 (2011).

9. Schlegel, K., Welte, C., Deppenmeier, U. & Muller, V. Electron transport during aceticlastic methanogenesis by *Methanosarcina acetivorans* involves a sodium-translocating Rnf complex. *FEBS J.* **279**:4444-4452 (2012).

10. Lane, D. in *Nucleic acid techniques in bacterial systematics* (eds E. Stackebrandt & M. Goodfellow) 115-175 (Jone Wiley&Sons, NY, 1991.

11. Luton, P.E., Wayne, J.M., Sharp, R.J. & Riley, P.W. The *mcrA* gene as an alternative to 16S rRNA in the phylogenetic analysis of methanogen populations in landfill. *Microbiology-(UK)*. **148**:3521-3530 (2002).

12. Geets, J., *et al*. DsrB gene-based DGGE for community and diversity surveys of sulfate-reducing bacteria. *J. Microbiol. Methods.* **66**:194-205 (2006).
